# The Nordic back pain subpopulation program: Can low back pain patterns be predicted from the first consultation with a chiropractor? A longitudinal pilot study

**DOI:** 10.1186/1746-1340-18-8

**Published:** 2010-04-29

**Authors:** Alice Kongsted, Charlotte Leboeuf-Yde

**Affiliations:** 1 The Nordic Institute of Chiropractic and Clinical Biomechanics, Forskerparken 10 A, 5230 Odense M, Denmark; 2 Spinecenter of Southern Denmark, Hospital Lillebaelt, Institute of Regional Health Research, University of Southern Denmark, Østre Hougvej 55, DK-5500 Middelfart, Denmark

## Abstract

**Background:**

It is widely believed that non-specific low back pain (LBP) consists of a number of subgroups which should be identified in order to improve treatment effects. In order to identify subgroups, patient characteristics that relate to different outcomes are searched for. However, LBP is often fluctuating or recurring rather than clearly limited in time. Therefore it would be relevant to consider outcome after completed treatment from a longitudinal perspective (describing "course patterns") instead of defining it from an arbitrarily selected end-point.

**Aims:**

The objectives of this pilot study were to investigate the interobserver reliability of a diagnostic classification system and to evaluate whether diagnostic classes or other baseline characteristics are associated with the LBP course pattern over a period of 18 weeks.

**Methods:**

Patients visiting one of 7 chiropractors because of LBP were classified according to a diagnostic classification system, which includes end-range loading, SI-joint pain provocation tests, neurological examination and tests for muscle tenderness and abnormal nerve tension. In addition, age, gender, duration of pain and presence of leg pain were registered in the patient's file. By weekly SMS-messages on their mobile phones, patients were asked how many days they had LBP the preceding week, and these answers were transformed into pain course patterns and the total number of LBP days.

**Results:**

A total of 110 patients were included and 76 (69%) completed follow-up. Thirty-five patients were examined by two chiropractors. The agreement regarding diagnostic classes was 83% (95% CI: 70 - 96). The diagnostic classes were associated with the pain course patterns and number of LBP days. Patients with disc pain had the highest number of LBP days and patients with muscular pain reported the fewest (35 vs. 12 days, p < 0.01). Men had better outcome than women (17 vs. 29 days, p < 0.01) and patients without leg pain tended to have fewer LBP days than those with leg pain (21 vs.31 days, p = 0.06). Duration of LBP at the first visit was not associated with outcome.

**Conclusions:**

The study indicated that there is a clinically meaningful relationship between diagnostic classes and the course of LBP. This should be evaluated in more depth.

## Background

Much has been written on non-specific low back pain (LBP) in the scientific literature. Presently, however, there are no easy answers to the clinicians' questions on how best to treat this condition; it seems that a number of different treatments have an effect, but only to a very limited degree [1-3]. In an attempt to break the stalemate, a number of researchers have shown an interest in the study of subpopulations of LBP [4-8] and preliminary results suggest that classification-based interventions are more effective than treatments directed towards mixed populations with non-specific LBP [9].

Different approaches exist to identify specific profiles of patients within the amorphous definition of non-specific LBP. Clinicians typically attempt to detect the pain generating structure and classify their patients accordingly into diagnostic subgroups. This information is then used both to determine the most relevant type of treatment and to predict outcome of treatment (prognosis). Such a pathoanatomical classification has also been suggested by some researchers [10] whereas others have focused on single clinical features [11,12] or clusters of characteristics that are predictive of response to treatment [7,13-16]. However, it is a challenging task to validate any classification system, as it would be necessary to test whether the base-line features actually make a difference to the outcome and if this difference is related to specific treatments. The latter would have to be done in randomized trials designed specifically for the purpose of subgroup identification [5].

In randomized trials, the outcome is typically calculated as the difference between the patients' status before and the status after treatment. However, LBP is a fluctuating or episodic condition for many [17-20]. These fluctuations occur even within a few months and have been shown to have varying patterns [19,20]. Therefore, it may not be relevant to measure outcome solely at one specific point in time, such as after 3 or 6 months, as there is no obvious end-point for LBP. However, presently this is how outcome of treatment for LBP is measured in clinical studies. A better outcome measure would rather be one that takes into account the course of pain over the post-treatment surveillance period.

Presently, very little is known of what happens between the time, when a patient seeks care, and when the final outcome is measured. However, with the advent of a new method to collect data using mobile phones, study subjects can be surveyed at frequent and regular intervals with the help of automatically generated text messages. This makes it possible to identify course patterns rather than end-point outcome thus approaching this problem from a different angle. Both diagnostic subgroups and other clinical characteristics could be held up against the clinical course, in order to see if they represent clinically relevant subgroups. After all, what matters for the patient is probably rather the every-day events than the arbitrarily selected point of outcome 3, 6 or 12 months after treatment took place.

For these reasons, a practice-based pilot study was performed, in which clinical data were collected at base-line and over a period of 18 weeks as continuous follow-ups by means of weekly text-messages. The rationale for the study was that clinical observations at the first consultation for an event of LBP would predict the ensuing course pattern. We have previously reported that improvement occurred early in the course [21] and that different course patterns existed within this study population of patients who were treated by chiropractors for a new event of LBP [20]. The objectives of the present report are 1) to get a feel for the inter-observer reliability of a diagnostic classification system [10], and 2) to investigate whether patients with different clinical profiles have different course patterns or different prognoses in terms of number of LBP days over a period of 18 weeks.

## Methods

### The study procedure

The method of the study has been described elsewhere [20]. In brief, chiropractors in private clinics collected baseline data using a standardized physical examination protocol for patients with LBP. Based on the examination patients were sub-grouped according to a classification system (described below), and they were then followed over 18 weeks with help of SMS track, a text message data collection system [22].

Seven chiropractors were invited to participate in the study on the condition that they followed an instruction program and agreed to use a specific clinical procedure. The inclusion criteria for the patients were that they had LBP with or without sciatica as the main complaint, were 18 - 65 years old and that they had a mobile phone. Patients were not included if one of the following non-inclusion criteria was present: Previous back surgery, pregnancy, other significant musculoskeletal problems in addition to the LBP, or inability to read or speak Danish. Prior to inclusion patients received written and verbal information about the study. Chiropractors were free to choose the kind and duration of treatment they found appropriate in each case.

### Instructions to participating chiropractors

Prior to data collection, the participating chiropractors had been informed of the purpose of the study and the rationale for the diagnostic classification system by the first author. At a one-day workshop they had been instructed on the performance of the clinical tests and their interpretation, and this was practised. The first author then visited the participating clinics once to supervise their clinical procedures when they examined LBP patients and to discuss which diagnostic class each patient belonged to. Questions were then answered and any mistakes rectified. After a period during wgich the group had had the possibility to practice the classification system in their own clinic, an evening meeting was undertaken to discuss any remaining problems and uncertainties before starting the collection of data.

### The diagnostic classification system

As part of the patient history age, gender, and duration of present complaint were noted down in the patient file, and in addition the chiropractors interviewed the patient at their own initiative. After this, the patient had a physical examination following a standardised protocol to classify the case according to a slightly modified version of the classification system previously described by Petersen et al. [10].

This classification system outlines an algorithm involving mechanical loading strategies as described by McKenzie [23], five pain provocative tests for sacroiliac joint pain [24], muscle palpation, tests for abnormal nerve tension, and a neurological examination including straight leg raise, muscle test, tendon reflexes, and test for sense of touch. Thirteen classes are described in the original version of the classification system, one of which consists of three subclasses (Additional File 1).

The single elements were performed as described in the original classification system, but in contrast to the original description, the chiropractors were allowed to use more than one of the classes if a patient fulfilled the criteria for more than one. Moreover, we excluded the diagnostic classes "adherent nerve root syndrome" and "nerve root entrapment syndrome" since these classes did not seem clinically meaningful, and it had not been possible to evaluate their reliability due to few cases in the only pre-existing reliability study [25].

The chiropractors could use the result of the classification in this information to the patient, or inform about their findings as they used to, patients were hence not blinded from their diagnosis, as in the normal clinical situation.

### Reliability of diagnostic classes

Reliability was tested with pairs of two observers, either two chiropractors from the same clinic or one of the participating chiropractors and the first author. Both clinicians were present during both the history and the physical examination. The examination was performed by one chiropractor (examiner A) while the other was allowed only to observe (examiner B). Both filled in an examination sheet without discussing the case. The chiropractors took turns with the roles of examiner A and B at a sequence not described in advance.

### Follow-up procedure

Participants were sent weekly text messages, beginning on the Sunday following the first consultation. If by the ensuing Thursday there had been no response, a reminder was sent to them. Information used in the present report relates to the following questions sent by SMS:

Question 1. Using a number from 0 to 7, please answer how many days you have been bothered by your lower back this week.

Question 2. Using a number from 0 to 7, please answer how many days you have been off work because of your lower back this week. (Answer with X if you are not working)

The answers were automatically entered into a data file that was later used for the analysis.

### Variables of interest

#### Independent variables

The independent variables were: diagnostic class (10 categories), leg pain (yes/no), age (continuous variable), gender, and duration of LBP at the time of base-line (acute [1-7 days], sub acute [8 days - 3 months], or chronic [> 3 months]).

#### Outcome variables

The outcome variables were generated from data collected weekly by means of SMS during 18 weeks. Based on question 1, "LBP days", patients were divided into 13 course patterns describing their individual course during 18 weeks (Table 1) [20]. These course patterns had been decided upon prior to the data analysis and without access to any clinical information about the participants. This has been described elsewhere [20]. Furthermore, "LBP days" was analysed as the total number of LBP days during 12 weeks since analyses of data from the entire 18 weeks would require replacement of inexpediently many missing values. Question 2 was analysed similarly as the total number of days with sick-leave during 12 weeks.

**Table 1:** Distribution of the defined course patterns in 78 patients with LBP (n) [20].

			5^th ^to 18^th ^week	
**At the 4th week**	**mainly recovered**	**stays in the initial category**	**moves - towards mainly improved**	**fluctuating**

Improved	**11**	**31**	NA	**7**

Unchanged	3	2	**6**	**12**

Worsened	0	1	0	4

(missing)	1	0	0	0

The five course patterns including at least 6 patients (marked with **bold **numbers) were used for the analysis of associations between baseline characteristics and course patterns, whereas patterns with fewer patients were pooled.

### Data analysis

Agreement regarding the diagnostic classes was evaluated both as agreement regarding the main diagnostic class (level 1) and in relation to all chosen classes (level 2). The more manifest diagnoses (in the order nerve root compression, spinal stenosis, disc pain) were given higher priority in relation to defining the main class in level 1 than the other classes. Dysfunction, postural syndrome and SI-joint pain ranked higher than facet-joint pain, abnormal nerve tension, muscle pain, and abnormal pain syndrome. Agreement was only calculated as percentages since there were too few observations to calculate meaningful kappa-values. In case of disagreement between examiners, we used examiner A's classification for the analyses of the main study.

The study population consisted of patients who participated at least until the 12^th ^week with no more than two weeks' pause in a row. Missing values during weeks 1 - 12 were replaced by the mean of the adjacent values from the week before and after the one missing. Due to the relatively small number of patients included in the pilot study, the three disc classes described in the classification system were collapsed into one as were pain course patterns consisting of less than six persons.

The analyses were done in two stages. First, each of the independent variables was tested against each of the outcome variables by Fisher's exact test or regression analysis with one explanatory variable. Thereafter, the variables that were associated with one of the outcome variables were considered for a multivariable analysis, providing that these associations had a p-value of less than 0.1. The multivariable analyses were performed by means of regression with robust variance estimations with LBP days as dependent variable. Results were considered statistically significant if p-values were below 0.05. The statistical package STATA 10.1 (StataCorp, Texas, USA) was used for the analyses.

## Results

### Descriptive data

Seven chiropractors (all women, average 7.6 years of clinical experience) from five chiropractic clinics in Denmark included participants for the study. Six of these chiropractors had graduated from the University of Southern Denmark and one from Palmer College, California, USA.

**Figure 1:**
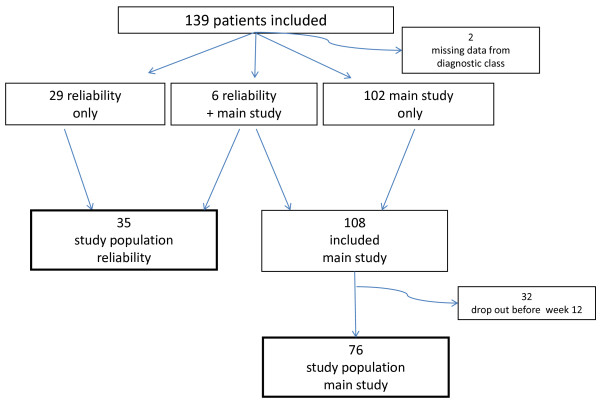
**Flow of 139 low back pain patients included for the study**.

A total of 139 patients (62 women; 77 men) underwent a physical examination in the project. The data from the examination was missing in two cases, 108 patients participated in the longitudinal study, and 29 were included only for the reliability study (Fig. 1). From the participants in the longitudinal study, 76 provided sufficient follow-up data to be used in the analysis of the present study. This population consisted of 38 men and 38 women with a median age of 41 years. Acute, sub-acute or chronic LBP was reported by 46%, 34%, and 20% respectively. Leg pain was present in 42% at inclusion.

### Diagnostic classification

The classification protocol was tested by two examiners in 35 patients (18 males; 17 females). The conclusions of each examiner appear in Additional file 2. Agreement regarding the most manifest diagnosis was obtained in 29 patients (83% [95% CI: 70-96%] agreement), whereas perfect agreement on both main class and eventually a second class was obtained in 19 patients (54% [95% CI: 37-72%] agreement). The most frequent diagnostic classes were lumbar dysfunction and disc related pain in the entire population as well as in the 76 patients constituting the study sample (Table 2). The distribution across classes differed between males and females (p = 0.01) (Table 2).

**Table 2:** Results of the diagnostic classification in a practice based study with 7 chiropractors.

Primary diagnostic class	Number (%) n = 137	% of male patients n = 76	% of female patients n = 61	Number (%) study population n = 76
Lumbar dysfunction	44 (32)	39	23	25 (32)

Disc pain	38 (27)	23	32	22 (29)

SI-joint pain	23 (17)	10	24	15 (20)

Facet joint pain	9 (6)	8	5	3 (4)

Muscle pain	7 (5)	6	3	6 (8)

Nerve root compression	4 (3)	1	5	1 (1)

Postural syndrome	3 (2)	3	2	2 (3)

Inconclusive	5 (4)	4	3	2 (3)

Missing data	2 (1)	1	2	0

### LBP days

Nine different course patterns were identified (Table 1) [20]. The course patterns with less than 6 patients were pooled for the analyses. The median number of LBP days during 12 weeks was 23.5 days (interquartile range 12 - 41).

### Sick-leave

The majority of the study population (53%) did not report any days with sick-leave. The median number of days with sick-leave in the 34 patients with any sick-leave was 4 days (interquartile range 2 - 8). Due to small numbers this variable was not used in the analyses of prognostic factors.

### Is there an association between baseline characteristics and the course pattern of LBP or total number of LBP days?

#### Age

The median age within the five pain course patterns varied from 36 to 49 years with patients in the' improved-recovered' and 'improved-stayed so' groups being the youngest (p = 0.01) (Table 3). There was not a significant correlation between age and the total number of days with LBP.

**Table 3:** Associations between baseline parameters and LBP course patterns in 76 chiropractor patients

	Pain course pattern						
	Improved-recovered	Improved- stayed so	Improved-fluctuated	Unchanged-improved	Unchanged-fluctuated	Other patterns or missing	p-value
Gender (%)							**< 0.01**
Males	28	46	3	10	5	8	
Females	3	33	15	21	10	18	
							
Age (median [IQR])	36 [33-43]	36 [31-51]	49 [34-56]	47 [41-53]	49 [42-59]	30 [46-54]	**0.02**
							
Duration (%)							0.8
1 - 7 days	17	40	6	17	6	14	
- 3 months	15	46	12	12	8	7	
> 3 months	13	27	13	20	13	13	
							
Leg pain							0.1
Yes	3	43	10	23	10	11	
No	24	37	10	12	7	7	
							
Diagnostic class (%)							0.05
Disc	5	32	23	14	14	12	
SI-joint	21	43	7	7	7	15	
Dysfunction	20	48	0	28	4	0	
Muscle	33	17	17	0	17	16	
Other*	9	45	0	9	0	36	

* Classes with less than five patients pooled

#### Gender

A larger part of the female patients (31%) had a pain course pattern with unchanged pain in the first weeks as compared to males (15%) (Table 3), and women reported a higher number of LBP days than men did (Table 4).

**Table 4:** Associations between baseline parameters and number of LBP days during 12 weeks in 76 chiropractor patients

	Total LBP days	p-value
	(median [IQR])	
Male	17 [8-31]	**< 0.01**
Females	29 [21-48]	

Duration		0.4
1 - 7 days	23 [10-41]	
- 3 months	22 [12-39]	
> 3 months	28 [15-49]	

Leg pain		0.6
yes	31 [14-46]	
no	21 [12-30]	

Diagnostic class		**0.01**
Disc	35 [23-54]	
SI-joint	22 [12-30]	
Dysfunction	19 [8-41]	
Muscle	12 [6-36]	
Other*	15 [7-29]	

*Classes with less than five patients pooled

***Duration of LBP pain at baseline *** was not associated with the pain course pattern or the total number of LBP days (Tables 3 and 4).

#### Leg pain

Patients with leg pain were less likely to experience the course pattern 'improved-mainly recovered' than patients without leg pain (3% vs. 24%), and patients with leg pain tended to report more LBP days, but differences were not significant (Tables 3 and 4).

### Is there an association between the diagnostic classification and the course pattern of LBP or total number of LBP days?

The diagnostic classes were associated with both the pain course patterns and the total number of LBP days (Tables 3 and 4). The highest number of LBP days was reported by patients with disc pain (median 35 days) and the class with the lowest number of LBP days was muscle pain (median 12 days).

### Multivariable analysis

The number of LBP days was tested in a model including diagnostic class and gender. Both gender and the diagnostic class were significantly associated to the total number of LBP days (Table 5). The associations with course patterns were not tested in a multivariable model because of too few patients in each diagnostic class and course pattern.

**Table 5:** Result of multivariate analysis with total number of LBP days as outcome. n = 76

Total number of LBP days	Regression coefficient [95% CI ]	p-value
Diagnostic Class		**0.01**
Disc (reference cat.)		
SI-joint	- 19 [- 30; -8]	
Dysfunction	- 13 [- 24; -1]	
Muscle	- 16 [-32; 0.5]	
Other*	- 19 [-34; - 4]	
		**0.02**
Gender		
Female vs. male	11 [2; 20]	

* Combines classes with less than five patients including two patients registered as inconclusive.

## Discussion

This appears to be the first study to compare baseline characteristics of LBP patients to pain patterns generated by very frequent follow-ups over a period of time. Moreover, it was the first attempt to study whether the prognosis of primary care patients with LBP is related to diagnostic classes as defined by the classification system described by Petersen [10]. Although we had a relatively small study sample, and a large number of subgroups, it was still possible to obtain some useful information.

First, it appears that at least some of the diagnostic classes relate to the prognosis. Patients classified as having disc-related pain reported more pain days and were less likely to experience the pain course 'mainly recovered' than others. Patients with disc pain had on average between 13 and 19 more days with pain than patients with muscle pain, mechanical dysfunctions, or SI-joint pain. It would be relevant to investigate such differences in more depth including whether diagnostic classes differ not only regarding pain, but also in relation to activity of daily living or disability. If similar associations between diagnosis and prognosis are confirmed by other studies, the differences are large enough to be important to patients and indicate that this classification system makes a distinction between relevant subgroups of patients.

In accordance with previous studies [26] men had a better prognosis than women. They had fewer days with LBP in total, were more likely to undergo the course pattern 'mainly recovered', and seemed to have less fluctuating patterns than women. The present results suggest that this could be, at least partly, explained by the difference in diagnostic classes between men and women, since men were less often classified with disc pain than women were. In addition, age was related to outcome patterns in the way that young patients had a milder course than older. The present cohort was not large enough to explore in more detail whether certain pain patterns relate to each gender or certain age groups and this should be explored in larger studies. In accordance with previous cohort studies on chiropractor patients [15], but maybe surprising to many clinicians, the duration of the present LBP episode was not associated to any of the outcome measures.

Because of the small numbers within each diagnostic class, statistical testing in relation to agreement was unworkable and the agreement was therefore only evaluated in percentages that do not take into account agreement by chance. The agreement concerning the diagnostic classes was high when based on the most manifest class, and markedly lower if absolute agreement was demanded. However, we consider the obtained agreement sufficient for the classification to be meaningful. The reliability of the classification system was tested in a set up with two chiropractors being present at the same consultation. This could have introduced bias toward higher agreement, but was chosen to avoid an altered symptom response at the second examination. The same decision was made in earlier studies [25,27]. The agreement on all classes was high (54%) as compared to a previous study on this classification system [25] with 34% agreement. The main difference, between the methods of the previous study and our, was that we allowed the use of more than one of the diagnostic classes. In the original study the classes were described as mutually exclusive. Therefore, in our study, it was possible to use more classes instead of making a compulsory final choice between two seemingly relevant classes. This approach seems reasonable because pain can be generated from more than one structure. We are aware of studies concluding that disc pain very seldom coexists with facet joint or SI-joint pain [28,29] and that pain is not likely to originate from both facet- and SI-joints at the same time [29]. However, these studies included only few patients who were not recruited from primary care, and in our analyses only one class was included in the analyses, consistent with the intention of the classification system.

The main limitation of this pilot study was the relatively large drop out from follow-up. As discussed in previous papers [20,21] this was in line with other primary care studies in which patients were followed up less frequently [16,19]. Fortunately, baseline characteristics in those who dropped out resembled those of the compliant patients. We suppose that a more enthusiastic information strategy directed to the participating patients could have helped maintaining the interest of the patients.

As a consequence of the quite small cohort we chose to pool the three disc classes from the original classification system into one. This may limit the prognostic value of the classification since we did not distinguish between mechanically reducible and irreducible discs, i.e. pain that can be centralized and pain that cannot, which is known to be of predictive value [30-32].

In conclusion, our results suggest that different diagnostic classes have different pain courses and indicate that patients with different low back conditions can be identified through the physical examination. The next step will be to perform a large-scale practice based study with a sufficient number of patients to make it possible to include more of the diagnostic classes and evaluate prognosis within each of these.

## Competing interests

The authors declare that they have no competing interests.

## Authors' contributions

Both authors participated in the design of the study and drafting of the manuscript. AK instructed the chiropractors who included patients, collected the data and did the data analyses.

## Supplementary Material

Additional file 1 **Diagnostic Classes**. The table lists the classes of the original classification system and the classes used in this study. Click here for file

Additional file 2 **Agreement between observers**. Two chiropractors' conclusion and their agreement regarding diagnostic class examining 35 LBP patients. Click here for file
