# Supplementary material for: The Nordic back pain subpopulation program: Can low back pain patterns be predicted from the first consultation with a chiropractor? A longitudinal pilot study
Source: Chiropr Osteopat. 2010 Apr 29;18:8. doi: 10.1186/1746-1340-18-8 (PMC2868855; doi:10.1186/1746-1340-18-8)
Supplement: Additional file 1 — Diagnostic Classes. The table lists the classes of the original classification system and the classes used in this study. [file 1746-1340-18-8-S1.PDF]

Diagnostic Classes in the original classification system [9]	Classes used in the present study	Classes used for the analyses
Disc Syndrome		x
Mechanical reducible disc	x	
Mechanical irreducible disc	x	
Non-mechanical disc	x	
Adherent nerve root syndrome		
Nerve root entrapment syndrome		
Nerve root compression	x	
Spinal stenosis syndrome	x	
Zygapophysial joint syndrome	x (termed facet joint pain)	
Postural syndrome	x	
Sacroiliac joint syndrome	x	x
Dysfunction syndrome	x	x
Myofascial pain syndrome*	x (termed muscle pain)	x
Adverse neural tension syndrome*	x	
Abnormal pain syndrome*	x	
Inconclusive	x	

*can coexist with other classes in the original description
