# Supplementary material for: The Nordic back pain subpopulation program: Can low back pain patterns be predicted from the first consultation with a chiropractor? A longitudinal pilot study
Source: Chiropr Osteopat. 2010 Apr 29;18:8. doi: 10.1186/1746-1340-18-8 (PMC2868855; doi:10.1186/1746-1340-18-8)
Supplement: Additional file 2 — Agreement between observers. Two chiropractors' conclusion and their agreement regarding diagnostic class examining 35 LBP patients. [file 1746-1340-18-8-S2.PDF]

Examiner A	Examiner B	Number of patients with that combination of classifications
Perfect agreement		
Dysfunction	Dysfunction	8
Dysfunction<br>Muscular pain	Dysfunction<br>Muscular pain	3
Discogenic pain<br>Abnorme Nerve Tension<br>Muscular pain	Discogenic pain<br>Abnorme Nerve Tension<br>Muscular pain	1
Spinal stenosis	Spinal stenosis	1
Spinal stenosis<br>Discogenic pain<br>Muscular pain	Spinal stenosis<br>Discogenic pain<br>Muscular pain	2
Discogenic pain	Discogenic pain	1
Discogenic pain<br>Muscular pain	Discogenic pain<br>Muscular pain	2
Discogenic pain<br>SI-joint<br>Muscular pain	Discogenic pain<br>SI-joint<br>Muscular pain	1
Agreement regarding primary diagnosis		
Dysfunction<br>Muscular pain<br>Abnorme Nerve Tension	Dysfunction<br>Muscular pain	1
Dysfunction<br>Muscular pain	Dysfunction	1
Nerve root compression	Nerve root compression<br>Spinal stenosis	1
Dysfunction<br>Facet joint pain	Dysfunction	1
Nerve root compression<br>Discogenic pain<br>Muscular pain	Nerve root compression<br>Discogenic pain	1

Inconclusive	Inconclusive<br>Dysfunction	1
Dysfunction	Dysfunction<br>SI-joint	1
Dysfunction<br><br>Muscular pain	Dysfunction<br>Postural<br>Muscular pain	1
Nerve root compression	Nerve root compression<br>Dysfunction<br>Muscular pain	1
Disagreement		
Spinal stenosis<br>Muscular pain	Nerve root compression<br>Spinal stenosis	1
Discogenic pain	Facet joint pain	1
Discogenic pain<br>Muscular pain	Facet joint pain	1
Dysfunction<br>Muscular pain	Muscular pain	1
Discogenic pain	Dysfunction<br>Muscular pain	1
Discogenic pain<br>Muscular pain	SI-joint<br>Muscular pain	1
Muscular pain<br>Abnorme Nerve Tension	Dysfunction<br>Abnorme Nerve Tension	1
